# 吉非替尼治疗晚期非小细胞肺癌耐药后的进展模式分析

**DOI:** 10.3779/j.issn.1009-3419.2013.10.02

**Published:** 2013-10-20

**Authors:** 彬 王, 昕 张, 琳 林, 学志 郝, 湘茹 张, 峻岭 李, 远凯 石

**Affiliations:** 100021 北京，中国医学科学院北京协和医学院肿瘤医院内科，抗肿瘤分子靶向药物临床研究北京市重点实验室 Department of Medical Oncology, Cancer Institute/Hospital, Chinese Academy of Medical Sciences & Peking Union Medical College; Beijing Key Laboratory of Clinical Study on Anticancer Molecular Targeted Drugs, Beijing 100021, China

**Keywords:** 吉非替尼, 肺肿瘤, 耐药, 转移, Gefitinib, Lung neoplsams, Resistance, Metastasis

## Abstract

**背景与目的:**

观察表皮生长因子受体酪氨酸激酶抑制剂吉非替尼（Iressa）治疗晚期非小细胞肺癌（non-small cell lung cancer, NSCLC）获益后出现耐药的临床表现和进程。

**方法:**

回顾性分析了我院内科接受吉非替尼治疗的93例晚期NSCLC患者，有效或稳定超过6个月，腺癌94.6%，女性79.6%，不吸烟者80.6%。每2个月评估疗效，观察耐药出现的临床表现。

**结果:**

本组93例，中位服药时间16个月（8个月-70个月），用药时间超过2年占21.5%（20/93），超过3年8.6%（8/93）。耐药出现时的临床表现主要为胸腔内进展，占80%（72/90），其中原发病灶及术后断端复发进展占38.9%（35/90），肺内转移占51.1%（46/90），胸膜转移占25.6%（23/90）；颅内进展30%（30/90）；腹腔内进展15.6%（14/90）。

**结论:**

EGFRTKI治疗耐药后的进展在临床上表现为多样化，治疗后的预后不同，因此，需要密切的临床随访以期早期发现、及时处理。

## Abstract (en)

**Background and objective:**

Clinical observation was conducted on the resistance to epidermal growth factor receptor tyrosine kinase inhibitor (EGFR-TKI) gefitinib (Iressa) therapy for advanced non-small cell lung cancer (NSCLC) patients.

**Methods:**

Ninety-three NSCLC patients in our hospital, showing effective or stable condition after 6-month previous gefitinib therapy, were included in this investigation. Among the patients, 94.6% of them were suffering from adenocarcinoma. The percentage of female is 79.6%; the percentage of non-smoking is 80.6%. During the therapy period, follow-up was preformed every 2 months.

**Results:**

Among the 93 patients, median therapy time was 16 months (range: 8 to 70 months), and 21.5% (20/93) of them had received therapy for more than 2 years, while 8.6% (8/93) had received that for more than 3 years. The progression included 80% (72/90) for intrapleural progression, 38.9% (35/90) for primary tumor plus recurrence after sugary especially, 51.1% (46/90) for intrapulmonary metastasis, 25.6% (23/90) for pleural metastasis, 30% (30/90) for intracranial progression and 15.6% (14/90) for intraperitoneal progression.

**Conclusion:**

Resistance to EGFR-TKI shows diversification in clinical observation, therefore, close clinical follow-up is necessary for early attention and timely treatment.

吉非替尼是小分子表皮生长因子络氨酸激酶抑制剂（epidermal growth factor receptor tyrosine kinases inhibitor, EGFR-TKI）之一，目前广泛应用于晚期非小细胞肺癌的治疗。对于有*EGFR*敏感性突变（外显子19缺失突变及外显子21点突变）的晚期非小细胞肺癌患者，EGFR-TKI的有效率及无进展生存时间优于化疗。TKI类的药物在服用一段时间后都会出现耐药的发生，临床上表现为疾病的进展，包括原有病灶的增大及新病灶的出现。对于TKI耐药后的治疗，临床正在探索中。使用TKI后根据耐药进展的不同表现推荐相应的后续处理，例如对于孤立的颅内转移及骨转移，推荐继续原方案治疗的同时行局部治疗。这些治疗选择的不同符合肺癌的个体化治疗原则。现总结分析作者于2007年1月-2012年7月间经治的服用易瑞沙后临床获益并服药时间超过6个月的93例患者出现耐药后的临床表现。

## 1 资料与方法

### 1.1 一般资料

全组93例患者中男性19例、女性74例；年龄33岁-79岁，中位年龄57岁，30岁-39岁5例，40岁-49岁18例，50岁-59岁32例，60岁-69岁24例，70及70岁以上14例；患者均经病理或细胞学证实为晚期非小细胞肺癌，其中腺癌88例，腺鳞癌1例，非小细胞肺癌（未分型）4例；既往吸烟患者13例，不吸烟者75例，记录不详5例。

### 1.2 治疗方法

吉非替尼250 mg口服，每日1次，服用6个月，根据影像学复查病灶评价有效或稳定，申请易瑞沙（吉非替尼）慈善赠药，获批后入组，每2个月进行影像学复查，疾病进展医生建议中止治疗或中止取药后出组。

### 1.3 定义

用药时间：开始口服吉非替尼-出组时间；疾病进展部位：根据影像学检查结果；新发转移部位：口服吉非替尼治疗之前影像学检查、临床记录没有出现转移的部位。

### 1.4 统计学方法

采用统计学软件SPSS 17.0统计。

## 2 结果

### 2.1 用药时间

因慈善供药项目入组条件，本组患者用药时间均 > 6个月。全组93例患者，用药时间8个月-70个月，中位用药时间16个月，8个-12个月29例，占31.2%（29/93）；13个-24个月44例，占47.3%（44/93）；25个月-36个月12例，占12.9%（12/93）；37个月-48个月6例，占6.5%（6/93）；48个月以上2例，分别为51个月、70个月，占2.2%（2/93）（图 1）。

**1:**
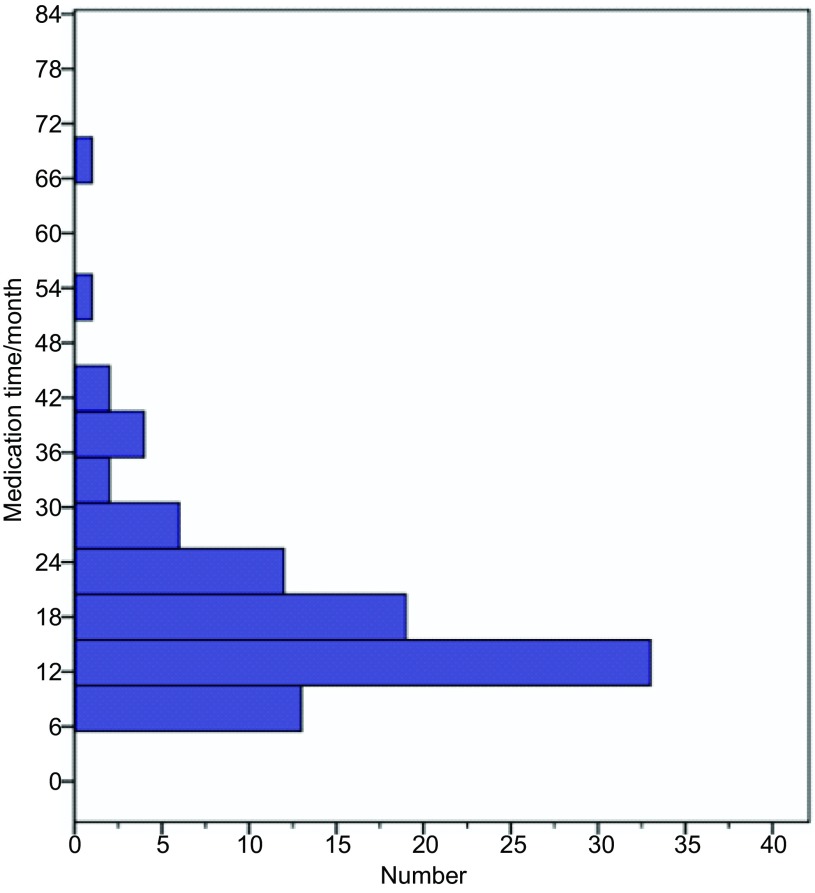
用药时间分布图 Time distribution of medication

### 2.2 出组情况

全组93例患者，2例由于脑梗（电话随诊）中止治疗出组，分别服药11个月、13个月，其中服药11个月患者出组前5个月脑核磁显示：点状强化灶警惕转移；服药13个月患者在吉非替尼疗前有脑转移经过放疗，颅内病灶稳定。另外1例患者用药51个月，自行中止继续用药。余90例患者均由于疾病进展，医生建议中止治疗出组。

### 2.3 疾病进展部位

90例患者出现疾病进展，胸腔内复发转移进展72例，占80%（72/90），包括原发灶进展33例，或术后断端的局部复发进展2例，或肺内转移进展46例，或胸膜转移进展23例，或肺门、纵隔、锁骨上淋巴结转移进展17例，或胸腔、心包积液进展15例；腹腔内转移进展14例，占15.6%（14/90），包括肝转移进展3例，或肾上腺转移进展4例，或腹膜后、腹腔淋巴结转移进展8例，或腹腔积液进展1例；颅内转移进展27例，占30%（27/90），包括疗前无颅内转移新发颅内转移16例，疗前既有颅内转移经过治疗复又出现进展或其他转移灶11例；骨转移进展9例，占10%（9/90）；远处浅表淋巴结转移进展3例，占3.3%（3/90），包括腋下淋巴结转移2例，腹股沟淋巴结转移1例（表 1）。

**1:** 吉非替尼耐药后进展部位与用药时间 Progress site and time of medication after resistance to gefitinib

Progress site	*n*	Percentage % (*n*/90)	Median medication time (months)
Intrathoracic lesions progress	72	80	16 (9-70)
Primary lesion	33	36.7	13 (9-30)
Postoperative broken ends	2	2.2	10, 25^*^
Intrapulmonary metastases	46	51.1	17 (9-43)
Pleural metastasis	23	25.6	17 (9-70)
Mediastinal, supraclavicular, and hilar LNM	17	18.9	13 (9-35)
Pleural and pericardial effusions	15	16.7	13 (9-28)
Intra-abdominal metastasis	14	15.6	13.5 (9-38)
Hepatic metastases	3	3.3	14, 21, 26^*^
Adrenal metastasis	4	4.4	11, 13, 17, 28^*^
Retroperitoneal abdomen LNM	8	8.9	12.5 (9-38)
Hydrops abdominis	1	1.1	13^*^
Intracranial metastatic	27	30	17 (8-40)
Bone metastasis	9	10	14 (8-70)
Superficial progress of LNM	4	4.4	9, 11, 13, 25^*^
Oxter LNM	3	3.3	11, 13, 25^*^
Groin LNM	1	1.1	9^*^
^*^The number is less than five, which ignore median medication time and just list the actual medication time. LNM: lymph node metastasis.			

## 3 讨论

吉非替尼作为EGFR-TKI之一治疗晚期非小细胞肺癌，10年间EGFR-TKI从临床研究中有效患者表现的临床特征^[1]^，到探索临床特征背后的分子学机制^[2, 3]^，经过印证，突变检测作为临床规范；通过临床的耐药表现，探索EGFR-TKI有效患者耐药的分子学机制^[4-8]^，同时，临床也将化疗与EGFR-TKI序贯^[9]^或联合或针对其他靶点的药物，意图控制肿瘤的不同克隆。肿瘤的个体化治疗是方向。临床医生的细致观察，是提出问题和假说的基础，基础研究回答问题挖掘临床表现背后深层次的原因，并需要通过临床来验证。

全组93例患者，女性占79.6%（74/93），明确病理类型腺癌占94.6%（88/93），病理无单纯的肺鳞癌，记录明确非吸烟者80.6%（75/93），符合EGFR-TKI治疗敏感人群的临床特征^[1]^。

吉非替尼治疗6个月稳定或有效的晚期非小细胞肺癌患者其后的随访中，中位用药时间16个月，用药时间超过2年占21.5%（20/93），用药不超过2年（包括2年）占78.5%（73/93）；超过3年8.6%（8/93），不超过占91.4%（85/93）。目前对于吉非替尼治疗晚期非小细胞肺癌的长期随访是每2个月进行影像学检查，参考上述数据，可考虑2年内的随访，依照原复查节奏，超过2年，尤其是3年后的随访可调整至3个月进行影像学的复查。

本组数据显示，吉非替尼耐药，胸腔内进展占80%（72/90），尤以原发灶+术后断端复发进展占38.9%（35/90），肺内转移占51.1%（46/90），胸膜转移占25.6%（23/90）；颅内进展30%（30/90）；腹腔内进展15.6%（14/90）。较高的中枢神经系统转移与其他研究类似^[10]^。随访过程中，参考上述数据，除根据已有病灶进行复查外，影像学检查常规可行：胸CT、颈部+腹部B-US，既往无颅内病灶者每6个月-12个月复查脑核磁。

综上所述，吉非替尼长期随访中可考虑2年内每2个月影像学复查，2年-3年后可每3个月影像学复查，复查包括胸CT、颈部+腹部B-US，6个月-12个月复查脑核磁。
